# Bimodal magnetic resonance and optical imaging of extracellular matrix remodelling by orthotopic ovarian tumours

**DOI:** 10.1038/s41416-020-0878-7

**Published:** 2020-05-11

**Authors:** Filip Bochner, Liat Fellus-Alyagor, Dafna Ketter, Ofra Golani, Inbal Biton, Michal Neeman

**Affiliations:** 1 Department of Biological Regulation, Weizmann Institute of Science, Rehovot, Israel; 2 Institute for Biomedical Engineering, ETH, Zürich Switzerland; 3 Life Sciences Core Facilities, Weizmann Institute of Science, Rehovot, Israel; 4 Department of Veterinary Resources, Weizmann Institute of Science, Rehovot, Israel

**Keywords:** Magnetic resonance imaging, Fluorescence imaging

## Abstract

**Background:**

The extracellular matrix modulates the development of ovarian tumours. Currently, evaluation of the extracellular matrix in the ovary is limited to histological methods. Both magnetic resonance imaging (MRI) and two-photon microscopy (2PM) enable dynamic visualisation and quantification of fibrosis by endogenous contrast mechanisms: magnetisation transfer (MT) MRI and second-harmonic generation (SHG) 2PM, respectively.

**Methods:**

Here, we applied the MT-MRI protocol for longitudinal imaging of the stroma in orthotopic human ovarian cancer ES-2 xenograft model in CD1 athymic nude mice, and for orthotopically implanted ovarian PDX using a MR-compatible imaging window chamber implanted into NSG mice.

**Results:**

We observed differences between ECM deposition in ovarian and skin lesions, and heterogeneous collagen distribution in ES-2 lesions. An MR-compatible imaging window chamber enabled visual matching between T2 MRI maps of orthotopically implanted PDX grafts and anatomical images of their microenvironment acquired with a stereomicroscope and SHG–2PM intravital microscopy of the collagen. Bimodal MRI/2PM imaging allowed us to quantify the fibrosis within the same compartments, and demonstrated the consistent results across the modalities.

**Conclusions:**

This work demonstrates a novel approach for measuring the stromal biomarkers in orthotopic ovarian tumours in mice, on both macroscopic and microscopic levels.

## Background

Ovarian cancer constitutes the deadliest gynaecological malignancy. According to the report of American Cancer Society of 2017, in the United States only, 22440 new cases of ovarian cancer were reported, and 14,080 women died.^1^ Standard treatment that involves debulking surgery and platinum-based chemotherapy remains inefficient as 80% of women relapse with drug-resistant disease and poor prognosis.^2^ Epithelial ovarian cancer causes extensive peritoneal disease that involves reactive tumour stroma—fibrotic, angiogenic and immunosuppressed niche shaped by reciprocal interactions of tumour cells, fibroblasts and the extracellular matrix.^3–5^ The ECM, that mainly consists of fibrillar proteins, such as collagen, as well as glucose–amino glycans (GAGs), fibronectin, laminins and proteoglycans,^6^ is not merely a scaffold for the cells embedded within, but plays an active role in modulating cell growth, differentiation, motility and viability.^7^ Deposited ECM provides support and anchorage for the proliferating tumour cells, blood vessels and stromal components. It was demonstrated in vivo that ovarian cancer cells can seed and breach the mesothelial barriers, attach to the underlying basement membrane and continue spreading collectively by utilising collagen fibres as a substrate for mesenchymal single-cell and collective migration.^8^ The collagen deposition in ovarian cancer was implicated in chemoresistance,^9^ and collagen-remodelling gene signature was found associated with metastasis and poor survival.^10^ Heavily fibrotic stroma may further obstruct delivery of therapeutics to the tumour.^11^

Typically, evaluation of fibrosis in the preclinical setting involves histopathological examination of affected tissues, which includes immunostainings for different ECM components, or chemical stains such as Sirius Red and Masson’s Trichrome. Such approaches, while indispensable in daily laboratory practice, require extensive tissue processing that affects the original tissue volume and may introduce staining artefacts. Both two-photon microscopy and MRI can be utilised to visualise fibrosis in vivo through distinct endogenous contrast mechanisms. Intravital 2PM enables high-resolution optical (~1 µm) imaging with penetration depth up to 1 mm.^12^ Use of nonlinear optical effects, such as frequency multiplication (second-harmonic generation), enables contrast-free imaging, as well as quantification of changes of collagen structure in ovarian cancer biopsies. It has been established that SHG signal reveals collagen density and directionality, both of which are biomarkers of disease.^13–15^ However, due to the limited field of view and depth of penetration, 2PM is restricted only to a minor fraction of a tissue. In contrast, MRI can be used to scan large tissue volumes, but at a lower resolution of 50–200 µm, which does not provide direct information about the tissue structure at the cellular level. MT described first by Stephen Wolf and Robert Balaban enables label-free imaging of fibrosis with MRI.^16^ It has been utilised to correlate the localisation of magnetisation transfer (MT) signal to the Picrosirius Red stainings in a chemically induced model of mammary carcinoma in rats^17^ and murine pancreatic xenograft models.^18^ Combination of label-free imaging with MR and 2PM can enable visualisation and quantification of tumour collagen at different scales. Localisation of unlabelled tumour tissue within the anatomical context of the microenvironment with optical approaches can facilitate accurate determination of specific regions in MRI images. Such approach may be useful for correlating intravital microscopy with clinical translatable MRI contrast mechanisms, and can aid in understanding the complex biology of tumour desmoplasia, as well as support the development of interventions directed towards the tumour stroma

Intravital optical imaging of orthotopic ovarian tumours in mice requires repeated exteriorisation of the ovary,^19^ or accessing the body cavity with laparoscopic objective lenses.^20^ Recently, we introduced surgically implanted imaging windows in order to perform longitudinal intravital imaging of the ovary, allowing us to dynamically record the attachment of ovarian cancer cells to the mesothelium, transmigration and spread on collagen fibres.^8^ Here, we demonstrate the longitudinal dual-modality imaging of collagen matrix remodelling in orthotopic ovarian xenografts, using MRI and 2PM microscopy. For that aim, we developed an MRI-compatible window that enables multimodal label-free intravital imaging both with MR and stereo- and 2PM. With this approach, we imaged fibrotic process in orthotopic ovarian tumours derived from human ovarian cancer cell lines as well as patient-derived xenografts, PDX (PDOX), implanted into immunocompromised murine transgenic strains.

## Methods

### Animals

All experiments were carried out according to Israel regulations on animal experimentation and Weizmann Institute guidelines. All experimental protocols were reviewed and approved by Weizmann Institutional Animal Care and Use Committee (IACUC), and were performed according to Israeli Legislation and Regulation on the Use of Animals in Biological and Medical Research.^21^

**Fig. 1:**
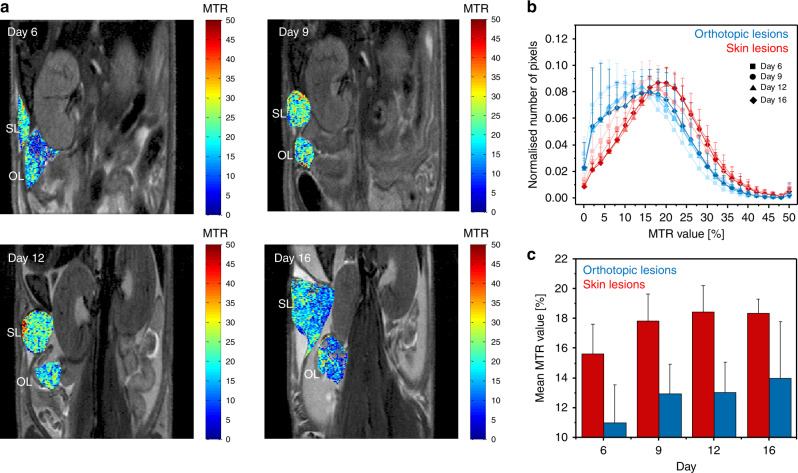
Longitudinal MT-MRI of collagen remodelling in ES-2 xenograft model. **a** T2-weighted MR images with MTR calculated within ROIs representing skin lesion (SL) and orthotopic ovarian lesion (OL). **b** Histogram of MTR values for skin lesion and orthotopic ovarian tumour normalised to the ROI size. **c** Mean MTR value calculated for each compartment and timepoint. Mixed model was fit to check for the influence of time on MTR, and the difference between the groups across all timepoints, both found significant with *p* < 0.01 (**) and *p* < 0.001 (***), respectively.

**Fig. 2:**
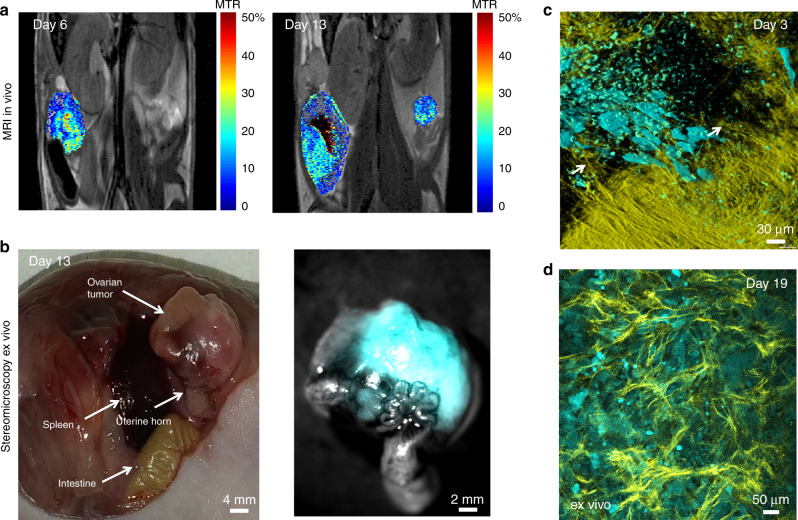
Tumour microenvironment heterogeneity underlying the variations in the MTR ratio. **a** MTR maps of the tumour area on days 6 and 13 after xenografting ES-2 cells (MRI in vivo). **b** Corresponding view of the same tumour within the anatomical context of the mouse abdomen post-mortem (stereomicroscopy ex vivo). Ovarian tumour with fluid-filled cyst encompassing the ovary, and part of the oviduct and uterine horn. **c** ES-2 cells imaged within de novo-forming collagen network in the mouse ovary (arrows) imaged throughout the imaging window with two-photon microscope 3 days post inoculation (Imaris rendering, MIP with 3D shading). The de novo-forming collagen fibres extend from the normal ovarian collagen. **d** The freshly excised tumour with reticuline network of collagen fibres, surrounding clusters of tumour cells. Cyan—tumour cells (eGFP), yellow—collagen (SHG).

Eight- to twelve-week CD1/nude mice (*n* = 9, Harlan, Israel) were used for longitudinal imaging of collagen with MR and 2PM in the xenograft model of ovarian cancer. Out of nine athymic mice, six were used for longitudinal experiment (Fig. 1), one for longitudinal experiment and necropsy (Fig. 2a, b) and two for imaging of collagen heterogeneity (Fig. 2c, d). Eight- to twelve-week Vecad/tdTomato mice (*n* = 3, Harlan, Israel) were used for syngeneic grafting of ID8 ovarian cancer cell line. Eight- to twelve-week NSG mice (*n* = 8, Harlan, Israel) were used for multimodal imaging of ovarian PDOX in MRI-compatible ovarian imaging window. Out of those four received tumour grafts, and the other four were used as unaffected controls.

All the animals from all experimental groups were kept under standard light cycle, received standard diet and were housed in individually ventilated cages with wood chip bedding and a cardboard shelter for enrichment. Their baseline health status was monitored for signs of distress or cachexia. None of those were observed throughout the experiments. All the procedures (see ‘Intravital imaging' and ‘Surgical procedures' for details) were performed in negative air-pressure barrier environment. All the animals were killed by cervical dislocation, while being under anaesthesia.

### Cell culture

ES-2-eGFP (ATCC, eGFP on the backbone of pIRES vector, under the EF-1a promoter) as well as ID8-GFP (transduced with retroviral vector carrying GFP,^22^ kindly provided by Dr. Kathrine Roby, University of Kansas Medical Center) lines were cultured in DMEM (Biological Industries, Beit Haemek, Israel) supplemented with 10% FCS and l-glutamine. At a confluence of 90%, they were harvested with 0.1% trypsin (Biological Industries, Beit Haemek, Israel) and washed with saline. Approximately 10 µl of pellet containing 3 × 10^6^ cells was injected into the ovarian bursa.

### Intravital imaging

The MRI and optical imaging were split into two imaging sessions, separated by a period of recovery of 5 h. Prior to MRI, animals were anaesthetised with IP injection of a mix of Domitor (Medetomidine, 1 mg/kg) and Ketamine (75 mg/kg), which provided stable sedation while minimising motion artefacts inside the scanner. Respiration rate was measured at 50–65 breaths per minute by using a monitoring system (Model 1025, SA Instruments, Stony Brook, NY). Once anaesthetised, the animals were placed in a head holder to assure reproducible positioning inside the magnet. Executing the imaging protocol took approximately 30 min per animal. Subsequently, the mouse was injected with Antisedan (1 mg/kg) and placed in the heated recovery unit (Termoplast, Italy). After the recovery period, the animals were anaesthetised again with Ketamine and Xylazine (i.p., Ketamine 0.1 μl/g body weight; Xylazine 0.1 μl/g body weight). Such mix was also used for optical imaging, even if the MRI has not been performed before. Throughout the imaging sessions with the stereo- and two-photon microscope, they were placed on the custom-imaging stage with feedback-controlled heating pad. The optical imaging session did not exceed to 40 min per animal.

### Stereomicroscopy

The images were acquired on MVX10 Microscope (Olympus, Japan), equipped with MV PLAPO 1× lenses (6.3–63×, NA 0.25, Olympus, Japan). KL-2500 LCD unit (Olympus, Japan) was used as a source of bright-field illumination. The system was coupled with PIXELFLY QE 12-bit CCD camera (PCO, Germany). The reflected light was passed through DM-505 filter (excitation 460–490 nm, emission 510–, longpass, Chroma, USA), to enhance the bright-field contrast due to increased haemoglobin absorption in the blood vessels.

### Two-photon microscopy

The images of collagen were acquired on LSM 880 Laser Scanning Microscope (Carl, Zeiss, Germany) with Plan Apochromat 20×/0.8 and 5×/0.16 lenses (Carl Zeiss, Germany) and Chameleon Ultra Ti:Sapphire laser (Coherent, USA). The imaging of PDX lesions was performed with 8 averages per line, at the excitation of 850 nm, with SHG signal detected at half the excitation wavelength in the range of 400–453 nm. Detector gain and laser intensity were set according to the amount of noise and fibrosis, and they were sample-dependent. The high-magnification stacks were acquired with 2 µm of z spacing, with resolution of 512 × 512 pixels and scaling of 0.83 µm/pixel. The low-magnification stacks consisted of 25 tiles with 10% overlap, acquired with resolution of 512 × 512 pixels, 50 µm of z spacing and scaling of 3.32 µm/pixel. The imaging of ID8-affected fat pads was performed with 16 averages per line with excitation of 880 nm, with SHG, GFP and tdTomato signal detected at 400–453 nm, 506–553 nm and 589–648 nm, respectively. The images consisted of 4–12 tiles with 12–30% overlap, and were acquired with 1024 × 1024 resolution with scaling of 1.67 µm/pixel.

### MRI

MRI experiments were performed on 9.4 Tesla BioSpec Magnet 94/20 USR system (Bruker, Germany) equipped with gradient coil system capable of producing pulse gradient of up to 40 gauss/cm in each of the three directions. All MR images were acquired with a 10-mm 1 H receive-only planar loop surface coil used in combination with a local pre-amplifier (Bruker, Germany) and transmitter linear coil (Bruker, Germany). The MRI protocol included T1-weighted images and two sets of T2-weighted RARE images (with and without MT module).

The T1-weighted images were acquired using the fast low-angle shot (FLASH) imaging sequence with the following parameters: a repetition delay (TR) of 123 ms, time echo (TE) of 2.9 ms, matrix dimension of 256 × 128 (interpolated to 256 × 256) and two averages, corresponding to an image acquisition time of 1 min 3 s. Five continuous slices (parallel to the chamber diameter) with slice thickness of 0.50 mm were acquired with a field of view (FOV) of 2.0 × 2.0 cm.

The two sets of multi-slice Rapid acquisition with relaxation enhancement (RARE) sequence (TR = 2200 ms, TE = 27 ms, RARE factor = 6) were used to acquire the same slice geometry as in the T1-weighted images. The two RARE sets were acquired with the MT module with the following parameters: one gauss pulse with amplitude of 0 μT (without MT irradiation) and 25 ΜT (with MT irradiation), duration of 10.96 ms, was applied 3 kHz off-resonance, four averages, corresponding to an image acquisition time of 3 min 4 s per set.

### Surgical procedures

#### Orthotopic implantation of tumours

The donor mouse was euthanised by cervical dislocation, and tumour was excised. Subsequently, it was cut into 2–3-mm pieces and maintained in Hanks’ Balanced Salt solution (HBSS, Thermofisher) on ice until implantation. The randomly selected recipient animals were anaesthetised with Ketamine and Xylazine (i.p., Ketamine 0.1 μl/g body weight; Xylazine 0.1 μl/g body weight) and placed on a heating pad. The right dorsolateral side was shaved and disinfected with 70% ethanol. Approximately a 5-mm long cut was made through all layers of the skin and separately through the peritoneum. The ovary was gently exteriorised outside of the body cavity and stabilised with a bulldog clamp holding the fat pad. Next, a small incision was made in the fat pad adjacent to the ovary. Surgical glue was applied onto the dissected tumour fragment, which was subsequently pressed into the incision. Alternatively, at this stage, the ES-2 cells were injected into the ovarian bursa, or ID8 cells were injected 12 days before mounting the imaging window. The ovary with the xenograft was gently pushed back inside the body cavity. The peritoneal membrane and the skin were stitched separately with absorbable coated Vicryl 2–0 suture and non-absorbable cotton suture (Ethicon, J&J), respectively. After surgery, animals were placed in the heated recovery unit (Termoplast, Italy).

#### Mounting of the imaging windows

The implantation of the imaging window was performed as described.^8^ Briefly, the mice were anaesthetised with Ketamine and Xylazine (i.p., Ketamine 0.1 μl/g body weight; Xylazine 0.1 μl/g body weight) and placed on a heating pad. The hair was removed with a shaver from the right dorsolateral side of the mouse, and the area was disinfected with 70% ethanol. A 12-mm round incision was made in the skin below the ribs, and the imaging window was positioned on top and subsequently stitched to the edge of the incision. Next, a small incision in the peritoneum was made below the tissue-supporting petals of the imaging window. The ovary was gently exteriorised from body cavity and immobilised on top of tissue-supporting petals with a surgical glue (Vetbond, USA). In the case of PDOX-bearing animals and ES-2 xenograft-bearing animals, in order to exteriorise the lesion with ease, an incision in the peritoneum was made before placing the imaging window. Only after establishing that the lesion is accessible, the imaging window was stitched to the skin. The animal was positioned on an in-house-made imaging stage, and the 13-mm-size coverslip, covered with sterile 1% hyaluronan gel (Biolon, Israel) was pushed into the groove and secured with a plastic C ring. Subsequently, the rest of the stitches were placed to attach the imaging window firmly to the skin. Finally, surgical glue was applied on the suture knots to prevent them from tearing. After surgery, the animals were placed in the heated recovery unit (Termoplast, Italy).

### Histology

Animals were sacrificed by cervical dislocation while still being anaesthetised after the last imaging session. The C ring and the cover glass were removed from the imaging window, and the tissue was gently detached from the supporting petals. The desired amount of tissue, corresponding to the accessible field of view in the imaging window, was dissected with scissors and attached to a cardboard in the same orientation as visible on the images, and placed in 4% PFA. Twenty-four hours later, the tissues were transferred to 1% PFA. Tissue dehydration and paraffinisation were performed with tissue processor TP 1050 (Leica, Germany), followed by embedment in paraffin with EG 1160 (Leica, Germany). In all, 4-µm sections were cut approximately from the middle of the tumour, so the ovary and fat pad would also be visible. The slides were automatically stained with TST-40 (Medite, Germany). The imaging was performed with Panoramic MIDI slide scanner (3D Histech, Hungary).

### Image analysis

#### Two-photon microscopy

The panoramic images (.lsm format) taken using 5× lenses were stitched using Zen 2 acquisition software and converted into maximum-intensity projections in Fiji and saved as .tiff. The images acquired with 20× lenses were loaded into Ilastik open-source software, where user-assisted image segmentation was performed to separate the collagen fibres from the background. The resulting 8-bit binary masks were subsequently analysed with Imaris (Bitplane, Switzerland). The surface was built based on the mask using absolute-intensity threshold, excluding voxels below 10 μm. The volume of the surface was calculated by Statistics module of Imaris. The thickness of the collagen layer was based on five manual measurements calculated per each tumour or fat pad. The two-photon stacks acquired in ID8-injected mice were stitched in Zen 2 software and saved as .tiff after exporting to Fiji.

#### Magnetisation transfer ratio

All image analyses were performed using purpose-written MATLAB (R2013B) scripts. The quantitative MTR maps were generated from the RARE images according to the following equation function on a pixel-by-pixel basis:

$${\mathrm{MTR}} = \frac{{\left( {100 \times I_{{\mathrm{without}}\,{\mathrm{MT}}\,{\mathrm{irradiation}}} - I_{{\mathrm{with}}\,{\mathrm{MR}}\,{\mathrm{irradiation}}}} \right)}}{{I_{{\mathrm{without}}\,{\mathrm{MT}}\,{\mathrm{irradiation}}}}}$$

The ovary, fat pad and tumour were manually segmented according to the stereomicroscopy images. Histograms of the MTR were calculated for fat pad and tumour regions per each slice. The mean MTR values of fat pad/tumour were calculated from the sum of MTR values of fat pad/tumour of all voxels in the ROI, and from all slices, and normalised by the sum of the number of pixels of fat pad/tumour from all slices.

### Statistical methods

Mixed model was fit to check for the influence of time on MTR, and the difference between the groups across all timepoints in the longitudinal MRI experiment. A Student’s t test was used to compare MTR means, collagen volume and thickness in the fat pad and tumour in the PDOX model. All the results were reported as mean ± SD, with *p* < 0.05 defining statistical significance (*), *p* < 0.01 (**), and *p* < 0.001 (***).

## Results

### Longitudinal imaging of collagen remodelling in ES-2 xenograft model with two-photon microscopy and MRI

**Fig. 3:**
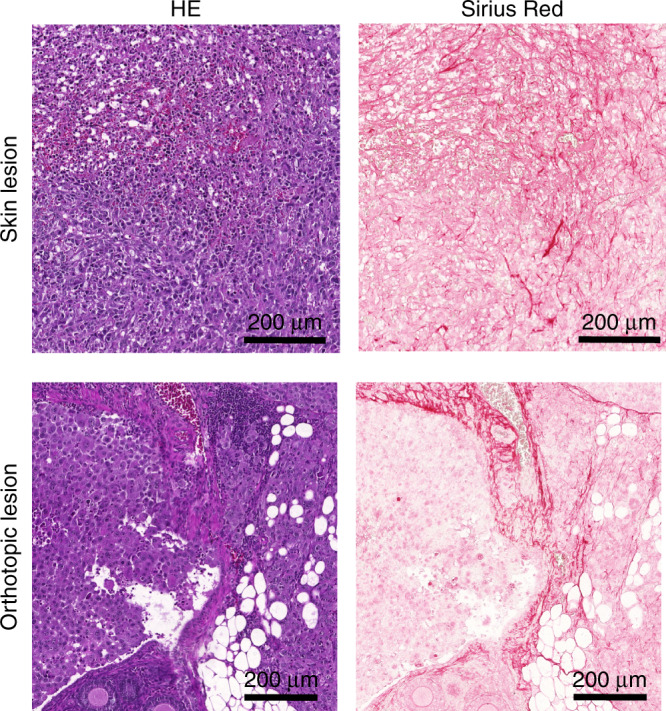
Histopathology of tumour stroma in ES-2 xenograft model 16 days post injection. HE and Sirius Red staining of the stroma.

To examine a longitudinal change in collagen content with MRI, human ovarian cancer ES-2 cells were xenografted into the ovarian bursa and imaged with MR 6, 9, 12 and 16 days after grafting. Tumour cells propagated in the ovary and fat pad areas, as well as in the surgical incision. MRI data were analysed by manual marking of the regions of interest (ROIs) according to anatomical cues visible on T2-weighted images (Fig. 1a; Supplementary Fig. 1), and MTR was calculated for the skin and ovarian lesion showing a longitudinal increase in MTR and difference in MTR between tumour sites. The skin lesion histogram showed higher MTR values (Fig. 1b). Likewise, such difference was reflected by mean MTR values (Fig. 1c; Supplementary Table 1). It should be noted that precise ROI determination in the abdomen is challenging; quantified ROIs likely contained regions of remodelled tissue, together with areas filled with fluid, heavily fibrotic as well as unaffected areas. For one of the mice imaged with MR 6, and 13 days upon grafting of ES-2 eGFP cells, we performed necropsy and subsequent optical imaging of the affected site. Longitudinal MRI revealed rapid growth of the lesion within the orthotopic site, which consisted of a hypointense region within the centre of the tumour and hyperintense rim (Fig. 2a). Necropsy revealed a large cyst filled with fluid, with solid tumour formed in the fat pad (Fig. 2b). 2PM performed with the imaging window in the same ovarian cancer model 3 days post grafting, revealed intermediate stages of stroma formation, where collagen network seeded by tumour cells was forming de novo over the normal ovarian surface (Fig. 2c). Necropsy and ex vivo two-photon imagining at day 19 post grafting revealed solid tumour tissue formed approximately 200 µm below the cover glass of the window (Fig. 2d), exhibiting the same collagen pattern as observed in Sirius Red-stained sections (Fig. 3). Similar observations of stromal heterogeneity were made in another ovarian cancer cell line. In syngeneic ovarian cancer model, ID8 cells implanted into Vecad/tdTomato mouse also remodelled the ovarian fat pad causing fibrosis and formation of vascular abnormalities. Twenty-four hours after imaging window implantation, 13 days post tumour grafting, thick bundles of collagen inhomogeneously distributed over the fat pad area were observed, contrasting the homogeneous distribution of collagen and blood vessels in the unaffected fat pad (Supplementary Fig. 2a, b). Sixteen days post grafting, ID8 tumours strongly remodelled the fat pad, forming niches where the SHG signal was strong, indicating a high degree of fibrosis, and those where the SHG signal was not detectable (Supplementary Fig. 2c). The macroscopic appearance of ID8 tumours differed from ES-2 tumours, ID8 lesions being more vascularised, and producing less-abundant ascites.

### Imaging fibrosis of ovarian PDOX in novel MRI-compatible optical imaging window

The MRI-compatible imaging windows were implanted 100 days after orthotopically xenografting the PDX into the ovarian fat pads. Twenty-four hours post surgery, MT-MRI was performed with surface coil placed directly on the imaging window, followed by stereomicroscopy and two-photon imaging of the collagen. Both PDX and control tissues were isolated for histopathological analysis.

**Fig. 4:**
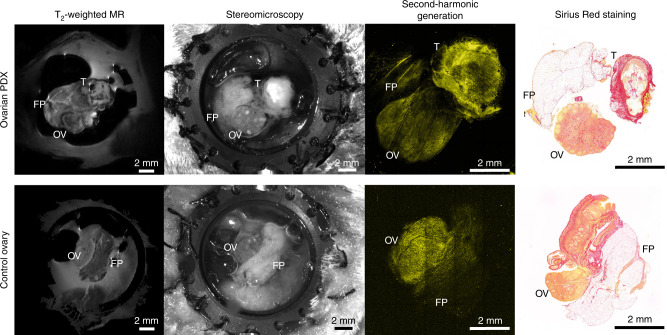
Visual matching between images acquired with MRI, stereomicroscope, two-photon microscope and the corresponding histological sections stained with Sirius Red. Optical access to the tissue is indispensable for finding the ROI for further quantification. The collagen distribution visible on in vivo two-photon images and on histological sections clearly represents the fibrotic character of the tumour lesion. FP fat pad, OV ovary, T tumour. Yellow—collagen (SHG).

The images acquired with a stereomicroscope facilitated understanding of tissue topology. The ovary could be recognised by the presence of follicles at different stages of development, as well as corpora albicans.^8^ The area of the tumour could be distinguished from the surrounding tissue due to vascularised rim and white core, or otherwise in some animals as an abnormally vascularised area nested in the fat pad. The interface between the tumour and the host fat tissue was well defined; the tumour tissue steeply transitioned into the normal fat pad. The panoramic two-photon images of collagen acquired at low magnification revealed the microstructure of the tissue. The collagen network in the tumour capsule produced a strong second-harmonic signal, whereas the signal emitted from the fat pad was scarce. T2-weighted images acquired with MRI gave a similar outline of tissue topography, but with a much lower resolution, making it difficult to distinguish between the tumour area and the adjacent fat pad. Histological evaluation of the imaged area upon Sirius Red staining revealed similar topology of the tissue components to in vivo images; the tumour capsule that surrounded the necrotic core was prominent and rich in collagen, whereas weakly stained fat pad lobules were enveloped in thin collagenous capsule. With the aid of optical imaging, it was possible to select more accurately the ROIs for quantification of the MT effect within the tumours. The tumour and control tissue imaged with multiple modalities in the same orientation are shown in Fig. 4.

**Fig. 5:**
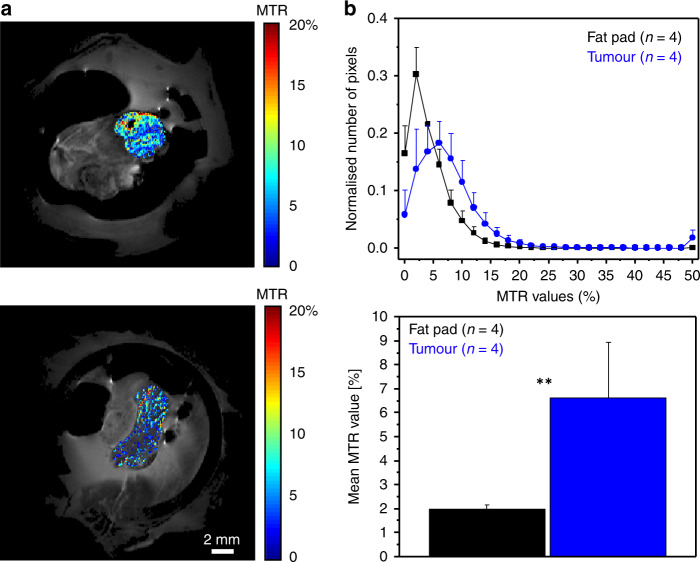
Quantification of MTR in PDOX and ovarian fat pad. **a** ROIs for calculation of MTR were chosen based on anatomical cues derived from optical imaging. The MTR was calculated pixel-by-pixel [MTR = (MT_off_–Mt_on_)/MT_off_], measured in percentage units. The colour bars were scaled to depict values in 0–50%. **b** Histogram of MTR values for tumour and fat pad, normalised to the ROI size, and calculation of the mean MTR value for both types of ROIs (*n* = 4 mice, respectively, two-tailed, unpaired Student's *t* test, *p* < 0.008).

**Fig. 6:**
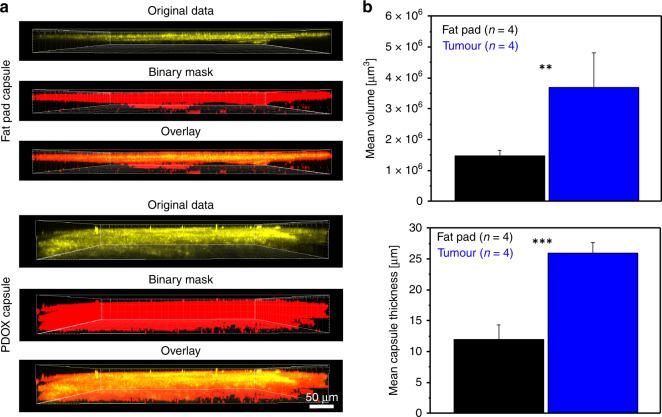
Two-photon imaging of PDOX and the native fat pad. **a** Orthogonal views of the collagen matrix inside the PDOX lesion and native fat pad. **b** Quantification of volume and capsule thickness based on the binary mask in PDOX and fat pad (*n* = 4 mice, respectively, two-tailed unpaired Student's *t* test; *p* < 0.008, *p* < 6.5E^−5^, respectively).

The MTR maps were calculated pixel-by-pixel for ROIs drawn on tumour lesions and fat pads separately with the aid of the images acquired with optical modalities (Fig. 5a). The mean MTR values inside the tumours were significantly higher than those in the fat pad, and they contained more pixels with elevated MTR (Fig. 5b). High-resolution, in vivo two-photon imaging of collagen was performed to evaluate the level of fibrosis in the tumour capsule (Fig. 6a). The collagen layer in the PDX had much higher thickness and volume than those in the ovarian fat pad (Fig. 6b).

To evaluate if PDOX develops tumours without the imaging window, we acquired MR images of the tumours in the orthotopic site, 64 days after grafting the PDX. We placed a block of 2% agarose next to the animal to demonstrate the sensitivity of our protocol to the hydrated macromolecules. The lesion was well separated from the surrounding tissue at this stage (Supplementary Fig. 3), which was confirmed by histological Sirius Red staining, which revealed the presence of collagenous capsule 79 days after implanting the tumours (Supplementary Fig. 3).

## Discussion

We have imaged longitudinally ES-2 xenograft lesions in nude mice, and detected differences in macromolecule levels in two compartments—the skin and orthotopic ovarian lesions. The skin, infiltrated by the tumour cells metastasising from the peritoneal cavity, presented higher macromolecule content that increased over time. Wound-healing process, initiated in the skin upon surgical incision, possibly facilitated the recruitment of tumour and stromal cells into the portal site, resulting in higher deposition of ECM proteins, which resulted in higher local MTR.^23^ The MTR within the measured ROIs was not uniform and contained hyper- and hypointense regions. As shown by 2PM here and in our previous study,^8^ the deposition of collagen in this model is not even, with collagen formed in de novo, unaffected and heavily remodelled areas. The ES-2 cells were shown to form undifferentiated carcinomas in mice that infiltrate the tissues and also form ascites.^24^ The ascitic fluid trapped within the tumours could also contribute to the heterogeneity of the signal. Similarly, in clinical picture, those tumours lack a gross architecture, being a mix of solid and cystic areas.^25^ Syngeneic engraftment of ID8 tumour cells into Vecad/tdTomato mice also caused ECM remodelling, leading to heterogeneous distribution of collagen I in the ovarian fat pad. Fibrosis in murine omentum was observed before in the same immunocompetent MOVCAR model, and additionally in xenograft models, such as HGSOC, OVCAR8 and OVCAR5, where secretion of collagen I was attributed to mesothelial cells through HIF1 and HIF2 pathways under hypoxic conditions.^26^ Thus, the presence of multiple fibrotic niches might be caused by hypoxia and heterogeneous infiltration of ECM-modifying cells, such as mesothelial cells and cancer-associated fibroblasts in the tumour microenvironment.

In order to enable MR and optical imaging of the ovarian PDX, we designed and constructed imaging windows manufactured from PEEK (polyether ketone), a biocompatible material, which opposite to metal alloys, does not generate susceptibility artefacts in the scanners,^27^ and is also radiolucent, which potentially enables introduction of CT alongside MRI and microscopy. We have utilised this method before for facilitated MRI of the pancreatic tissue.^28^ Here, based on wide-field microscopy, we were able to locate the lesions within the anatomical context accurately, and mark ROIs accordingly on the corresponding T2 maps. Anatomical context helped to visually match the SHG signal of collagen type I and macromolecule signal and quantify it in tumour implanted into ovarian fat pads. This site was chosen for grafting, as regardless of the exact origin of ovarian cancer, being ovarian mesothelium, fallopian tube or the gastrointestinal tract,^29^ ovarian cancer cells were demonstrated to home to the ovarian fat pad in the murine preclinical models.^8,30^ Even when injected directly into the ovarian bursa, the tumours encompass the fat pad, ovary and uterine horn (as shown in Fig. 2b), subsequently invading distant peritoneal sites. In patients, tumours home to the lipid-rich omentum.^31^ It was shown that adipocytes secrete interleukin-8 that attracts ovarian cancer cells that utilise fatty acids as a source of energy.^30^ In our study, PDX tumours were placed in the fat tissue proximal to the ovary. Since they were part of the same microenvironment as the ovary, and grew in a favourable, clinically relevant place, their grafting location constituted the orthotopic site. Implanting PDX fragments straight into the ovary that is 2–3-mm size, could damage the organ early, and prevent tumours from developing rich vascular supply, since the area of contact between tissues would be insufficient. The PDX model we used in the study originated from primary malignancy, and it was defined as stage III papillary serous adenocarcinoma. We observed that in murine fat pad, the lesions were well-separated from the surrounding tissue by the collagen capsule, which was confirmed by Sirius Red staining on tissue sections. MTR and SHG signal were increased within the same, matching, collagen-rich areas. Lack of peritoneal metastatic disease, the presence of collagenous capsule in NSG mice, area-specific increase in SHG and macromolecule content constitute biomarkers of benign neoplastic disease. In humans, the presence of localised, well-encapsulated lesions, is associated with good prognosis.^32,33^

We have applied the MT-MRI protocol and 2PM to image tumour lesions in orthotopic ES-2 xenograft and PDX (PDOX) models in the MRI-compatible imaging window. T2 relaxation rate of protons associated with macromolecules is too short to be measured directly. MT-MRI is based on indirect measurement of macromolecule content in the tissue. To this end, the off-resonance pulse is applied, so the magnetisation in the protons associated with the macromolecules is quenched. The constant chemical exchange of protons between the water sheath surrounding the macromolecules and the bulk water, enables restoration of the magnetisation in the sheath, which causes a proportional decrease of magnetisation in the bulk, which can be measured directly. The change of the signal in the bulk water can be expressed as magnetisation transfer ratio (MTR), which is proportional to the amount of macromolecules in the tissue.^34^ MT-MRI is used in the evaluation of liver,^35^ renal^36^ and bowel fibrosis,^37^ multiple sclerosis,^38^ osteoarthritis,^39^ atherosclerosis^40^ and other conditions that are characterised by alterations of macromolecule content in the tissue. Fibrillar collagen, deposited by tumour-associated fibroblasts, constitutes a major component of the tumour stroma. It has been shown ex vivo^41^ and in vivo^18^ that it can give rise to MT signal, and it is abundant in the stroma of preclinical cancer models and in patients.^42^ Ovarian cancer ECM consists of collagen IV, fibrillar collagens, hyaluronan and heparan sulfate proteoglycans (extensively reviewed^42^), and although the fibrillar collagen type I distribution visualised with SHG contrast was spatially correlated to MT signal, it is possible that other ECM proteins also contributed to the macromolecule signal in imaged tumour models as well.

MRI is commonly used among other methods, such as ultrasound and computerised tomography (CT), to evaluate the nature of the ovarian cancer mass.^43,44^ It can be used to distinguish between benign and malignant ovarian tumours with the accuracy of 88–92%.^45^ Although CT constitutes the first line of ovarian cancer detection and standard of care for preoperative evaluation, it exposes patients to radiation,^46^ which limits its usefulness for longitudinal follow-up of the patients. It has been shown that MRI sequences have the potential to measure clinical chemotherapy-response biomarkers,^47^ and aid preoperative assessment of lesions.^48^ Multiparametric MRI can differentiate between benign and malignant lesions.^49^ Therefore, imaging of ovarian cancer biomarkers with MRI simultaneously with other modalities in the preclinical setting constitutes an important step towards expanding MRI potential in the clinic.

MTR used to characterise ovarian cancer lesions in this study, constitutes a clinically relevant biomarker of fibrosis. Desmoplastic changes in the tumour microenvironment are common in many cancers metastasising into the peritoneal cavity, including pancreatic, liver, ovarian and colon cancer. The clinical potential of MT-MRI in assessing fibrosis in the heterogeneous tumour microenvironment has been shown in the clinical setting. Chemotherapy induced changes in breast tumours, and reproducibility of measurements across time and scanners has been demonstrated; it was shown that areas with high fibrosis are associated with high MTR, and oedematous areas are associated with low fibrosis,^50^ which is congruent with our observations about tumour heterogeneity in preclinical ovarian cancer models. Similarly, MTR has been used to identify fibrosis in patients with rectal cancer after radiotherapy, demonstrating high MTR values for fibrosis in residual tumour, and lower values for oedematous and normal tissues, demonstrating diagnostic potential of the method.^51^ Although MTR depends on pulse sequence and magnetic field strength, and it is not standardised between the scanners, it can be easily employed clinically as MT sequences are available on modern scanners, and they do not require additional hardware or software.^52^ MTR imaging could accompany standard MRI protocols used to diagnose and classify the lesions, provide information about the fibrotic status of the tumour, without the need for contrast-agent injection and aid patient stratification as the presence of fibrotic gene expression signature has been shown to predict poor response to the chemotherapy.^53^ Since MRI is commonly used to non-invasively differentiate between the benign and malignant ovarian cancer lesions, our preclinical observations encourage to further explore MTR as a fibrosis biomarker in clinical ovarian cancer imaging.

The study also has an impact on reduction and refinement aspects of animal use in research. Non-invasive longitudinal imaging of fibrotic stroma helped to reduce the number of animals used in experiments while providing insights into a clinically relevant biomarker of disease—the macromolecule content with particular contribution of collagen type I, which was implicated in the desmoplastic reaction contributing to the therapy resistance. Thanks to a dual window, fibrosis could be quantified both at micro- and mesoscale, with different imaging modalities in one animal. Patient-derived xenografts (PDX) were introduced in order to improve the recapitulation of the human disease in mice, and the effectiveness of interventions tested in preclinical research; thus, measured properties of tumour stroma in the PDOX model we used in the study, are likely to be present in human disease. In the preclinical setting, the imaging approach we have developed has a potential to be used for testing the effects of anti-fibrotic and anti-angiogenic treatments utilising novel antibodies, small-molecule inhibitors, drug carriers and cell-based therapies involving fibroblasts or immune cells. Since the method enables longitudinal imaging, basic biological questions regarding the influence of hormones on tumour perfusion, fibrosis or tumour cell migration could be addressed. Correlating microanatomical parameters, i.e., collagen density, structure or blood vessel morphology, with MRI contrast such as BOLD and DW-MRI, could further aid in finding their biological origin in preclinical cancer models.

In summary, we report here a novel approach to image fibrosis within orthotopic ovarian tumours in vivo. Bimodal, contrast-free visualisation of tumour stroma with MRI and 2PM enabled by the novel imaging window constitutes an unexplored approach towards the evaluation of disease biomarkers in the preclinical setting. Although integrated MRI and 2PM approaches have been demonstrated before ex vivo,^54^ bimodal intravital imaging through the MR-compatible window can be utilised when imaging modalities are separated in space as in most preclinical imaging facilities. Such approach may provide novel insight into peritoneal pathologies such as ovarian cancer, and shed light on altered tissue properties within tumour stroma occurring both at macroscopic and microscopic levels.

## Supplementary information

Supplementary Information

## Data Availability

The data sets used and/or analysed during this study are available from the corresponding author on reasonable request, and most of the original data are included in this paper.

## References

[1] Siegel RL, Miller KD, Jemal AJCacjfc (2017). Cancer statistics, 2017. CA: Cancer J. Clin..

[2] Davis A, Tinker AV, Friedlander MJGo (2014). “Platinum resistant” ovarian cancer: what is it, who to treat and how to measure benefit?. Gynecol. Oncol..

[3] Helleman J, Jansen MP, Burger C, van der Burg ME, Berns EMJTijob, biology c (2010). Integrated genomics of chemotherapy resistant ovarian cancer: a role for extracellular matrix, TGFbeta and regulating microRNAs. Int. J. Biochem. Cell Biol..

[4] Zhang Y, Tang H, Cai J, Zhang T, Guo J, Feng D (2011). Ovarian cancer-associated fibroblasts contribute to epithelial ovarian carcinoma metastasis by promoting angiogenesis, lymphangiogenesis and tumor cell invasion. Cancer Lett..

[5] Moran-Jones K, Gloss BS, Murali R, Chang DK, Colvin EK, Jones MD (2015). Connective tissue growth factor as a novel therapeutic target in high grade serous ovarian cancer. Oncotarget.

[6] Badylak SF (2007). The extracellular matrix as a biologic scaffold material. Biomaterials.

[7] Lu P, Weaver VM, Werb Z (2012). The extracellular matrix: a dynamic niche in cancer progression. J. Cell Biol..

[8] Bochner F, Fellus-Alyagor L, Kalchenko V, Shinar S, Neeman M (2015). A novel intravital imaging window for longitudinal microscopy of the mouse ovary. Sci. Rep..

[9] Sherman-Baust CA, Weeraratna AT, Rangel LB, Pizer ES, Cho KR, Schwartz DR (2003). Remodeling of the extracellular matrix through overexpression of collagen VI contributes to cisplatin resistance in ovarian cancer cells. Cancer Cell..

[10] Cheon D-J, Tong Y, Sim M-S, Dering J, Berel D, Cui X (2014). A collagen-remodeling gene signature regulated by TGF-β signaling is associated with metastasis and poor survival in serous ovarian cancer. Clin. Cancer Res..

[11] Netti PA, Berk DA, Swartz MA, Grodzinsky AJ, Jain RK (2000). Role of extracellular matrix assembly in interstitial transport in solid tumors. Cancer Res..

[12] Theer P, Hasan MT, Denk W (2003). Two-photon imaging to a depth of 1000 µm in living brains by use of a Ti:Al_2_O_3_ regenerative amplifier. Opt. Lett..

[13] Nadiarnykh O, LaComb RB, Brewer MA, Campagnola PJJBc (2010). Alterations of the extracellular matrix in ovarian cancer studied by second harmonic generation imaging microscopy. BMC cancer.

[14] Kirkpatrick ND, Brewer MA, Utzinger UJCE, Biomarkers P (2007). Endogenous optical biomarkers of ovarian cancer evaluated with multiphoton microscopy. Cancer Epidemiol. Prev. Biomark..

[15] Adur J, Pelegati VB, de Thomaz AA, Baratti MO, Almeida DB, Andrade L (2012). Optical biomarkers of serous and mucinous human ovarian tumor assessed with nonlinear optics microscopies. PLoS ONE.

[16] Wolff SD, Balaban RSJMrim (1989). Magnetization transfer contrast (MTC) and tissue water proton relaxation in vivo. Magn. Reson. Med..

[17] Jerome NP, Boult JK, Orton MR, d’Arcy JA, Nerurkar A, Leach MO (2018). Characterisation of fibrosis in chemically-induced rat mammary carcinomas using multi-modal endogenous contrast MRI on a 1.5 T clinical platform. Eur. Radiol..

[18] Li W, Zhang Z, Nicolai J, Yang GY, Omary RA, Larson ACJMrim (2012). Magnetization transfer MRI in pancreatic cancer xenograft models. Magn. Reson. Med..

[19] Watson JM, Marion SL, Rice PF, Bentley DL, Besselsen DG, Utzinger U (2014). In vivo time-serial multi-modality optical imaging in a mouse model of ovarian tumorigenesis. Cancer Biol. Ther..

[20] Williams RM, Flesken-Nikitin A, Ellenson LH, Connolly DC, Hamilton TC, Nikitin AY (2010). Strategies for high-resolution imaging of epithelial ovarian cancer by laparoscopic nonlinear microscopy. Transl. Oncol..

[21] Kalman, R., Harmelin, A., Ziv, E. & Fischer, Y. Israeli legislation and regulation on the use of animals in biological and medical research. in Javier Guillén editor. *Laboratory Animals* 203–219 (Elsevier, 2018).

[22] Zhang L, Yang N, Garcia J-RC, Mohamed A, Benencia F, Rubin SC (2002). Generation of a syngeneic mouse model to study the effects of vascular endothelial growth factor in ovarian carcinoma. Am. J. Pathol..

[23] Ataseven B, Grimm C, Harter P, Heikaus S, Heitz F, Traut A (2016). Prognostic impact of port-site metastasis after diagnostic laparoscopy for epithelial ovarian cancer. Ann. Surg. Oncol..

[24] Shaw TJ, Senterman MK, Dawson K, Crane CA, Vanderhyden BCJMt (2004). Characterization of intraperitoneal, orthotopic, and metastatic xenograft models of human ovarian cancer. Mol. Ther..

[25] Kennedy AW, Biscotti CV, Hart WR, Webster KDJGo (1989). Ovarian clear cell adenocarcinoma. Gynecol. Oncol..

[26] Natarajan S, Foreman KM, Soriano MI, Rossen NS, Shehade H, Fregoso DR (2019). Collagen remodeling in the hypoxic tumor-mesothelial niche promotes ovarian cancer metastasis. Cancer Res..

[27] Bennett L, Wang P, Donahue MJJoap (1996). Artifacts in magnetic resonance imaging from metals. J. Appl. Phys..

[28] Martins AF, Clavijo Jordan V, Bochner F, Chirayil S, Paranawithana N, Zhang S (2018). Imaging insulin secretion from mouse pancreas by MRI is improved by use of a zinc-responsive MRI sensor with lower affinity for Zn2+ ions. J. Am. Chem. Soc..

[29] Mandai, M. Molecular pathogenesis of ovarian cancer: an inextricable maze. in Katabuchi H, Ohba T, Motohara T. editors. *Cell Biology of the Ovary*. 123–134 (Springer, 2018).

[30] Nieman KM, Kenny HA, Penicka CV, Ladanyi A, Buell-Gutbrod R, Zillhardt MR (2011). Adipocytes promote ovarian cancer metastasis and provide energy for rapid tumor growth. Nat. Med..

[31] Lengyel EJTAjop (2010). Ovarian cancer development and metastasis. Am. J. Pathol..

[32] Rakha EA, Gandhi N, Climent F, van Deurzen CH, Haider SA, Dunk L (2011). Encapsulated papillary carcinoma of the breast: an invasive tumor with excellent prognosis. Am. J. Surg. Pathol..

[33] Ng IO, Lai EC, Fan ST, Ng MMJC (1992). Tumor encapsulation in hepatocellular carcinoma. A pathologic study of 189 cases. Cancer.

[34] Henkelman R, Stanisz G, Graham SJNiB (2001). Magnetization transfer in MRI: a review. NMR Biomed..

[35] Fuchs BC, Wang H, Yang Y, Wei L, Polasek M, Schühle DT (2013). Molecular MRI of collagen to diagnose and stage liver fibrosis. J. Hepatol..

[36] Jiang K, Ferguson CM, Ebrahimi B, Tang H, Kline TL, Burningham TA (2016). Noninvasive assessment of renal fibrosis with magnetization transfer MR imaging: validation and evaluation in murine renal artery stenosis. Radiology.

[37] Dillman JR, Swanson SD, Johnson LA, Moons DS, Adler J, Stidham RW (2015). Comparison of noncontrast MRI magnetization transfer and T2‐weighted signal intensity ratios for detection of bowel wall fibrosis in a Crohn’s disease animal model. J. Magn. Reson. Imaging.

[38] Filippi MJN (1999). Magnetization transfer imaging to monitor the evolution of individual multiple sclerosis lesions. Neurology.

[39] Yao W, Qu N, Lu Z, Yang SJSr (2009). The application of T1 and T2 relaxation time and magnetization transfer ratios to the early diagnosis of patellar cartilage osteoarthritis. Skelet. Radiol..

[40] Qiao Y, Hallock KJ, Hamilton ,JA (2011). Magnetization transfer magnetic resonance of human atherosclerotic plaques ex vivo detects areas of high protein density.. J. Cardiovasc. Magn. R.

[41] Harel A, Eliav U, Akselrod S, Navon G (2008). Magnetization transfer based contrast for imaging denatured collagen. J. Magn. Reson. Imaging..

[42] Burleson KM, Casey RC, Skubitz KM, Pambuccian SE, Oegema Jr TR, Skubitz AP (2004). Ovarian carcinoma ascites spheroids adhere to extracellular matrix components and mesothelial cell monolayers. J. Gynecol. Oncol..

[43] Valentini AL, Gui B, Miccò M, Mingote M, De Gaetano A, Ninivaggi V (2012). Benign and suspicious ovarian masses—MR imaging criteria for characterization: pictorial review. J. Oncol..

[44] Jung SE, Lee JM, Rha SE, Byun JY, Jung JI, Hahn ST (2002). CT and MR imaging of ovarian tumors with emphasis on differential diagnosis. Radiographics.

[45] Bazot M, Daraï E, Nassar-Slaba J, Lafont C, Thomassin-Naggara I (2008). Value of magnetic resonance imaging for the diagnosis of ovarian tumors: a review. J. Computer Assist. Tomogr..

[46] Kitajima K, Murakami K, Sakamoto S, Kaji Y, Sugimura K (2011). Present and future of FDG-PET/CT in ovarian cancer. Ann. Nucl. Med..

[47] Deen SS, Priest AN, McLean MA, Gill AB, Brodie C, Crawford R (2019). Diffusion kurtosis MRI as a predictive biomarker of response to neoadjuvant chemotherapy in high grade serous ovarian cancer. Sci. Rep..

[48] Engbersen M, van’t Sant I, Lok C, Lambregts D, Sonke G, Beets-Tan R (2019). MRI with diffusion-weighted imaging to predict feasibility of complete cytoreduction with the peritoneal cancer index (PCI) in advanced stage ovarian cancer patients. Eur. J. Radiol..

[49] Türkoğlu S, Kayan M (2020). Differentiation between benign and malignant ovarian masses using multiparametric MRI. Diagn. Interv. Imaging.

[50] Virostko J, Sorace AG, Wu C, Ekrut D, Jarrett AM, Upadhyaya RM (2019). Magnetization transfer MRI of breast cancer in the community setting: reproducibility and preliminary results in neoadjuvant therapy. Tomography.

[51] Martens MH, Lambregts DM, Papanikolaou N, Heijnen LA, Riedl RG, zur Hausen A (2014). Magnetization transfer ratio: a potential biomarker for the assessment of postradiation fibrosis in patients with rectal cancer. Investig. Radiol..

[52] Winfield J, Payne G, Desouza N (2015). Functional MRI and CT biomarkers in oncology. Eur. J. Nucl. Med. Mol. Imaging.

[53] Batista L, Gruosso T, Mechta-Grigoriou F (2013). Ovarian cancer emerging subtypes: role of oxidative stress and fibrosis in tumour development and response to treatment. Int. J. Biochem. Cell Biol..

[54] Cui M, Zhou Y, Wei B, Zhu X-H, Zhu W, Sanders MA (2017). A proof-of-concept study for developing integrated two-photon microscopic and magnetic resonance imaging modality at ultrahigh field of 16.4 tesla. Sci. Rep..

