# Supplementary material for: Bimodal magnetic resonance and optical imaging of extracellular matrix remodelling by orthotopic ovarian tumours
Source: Br J Cancer. 2020 May 11;123(2):216–25. doi: 10.1038/s41416-020-0878-7 (PMC7374547; doi:10.1038/s41416-020-0878-7)
Supplement: Supplementary file 1 — Supplementary Information [file 41416_2020_878_MOESM1_ESM.docx]

**Bimodal magnetic resonance and optical imaging of extracellular matrix remodeling by orthotopic ovarian tumors.**

Filip Bochner^1^, Liat Fellus-Alyagor^1^, Dafna Ketter^1^, Ofra Golani^2^, Inbal Biton^3^ and Michal Neeman^1^

^1^Department of Biological Regulation, ^2^Life Sciences Core Facilities, and ^3^Department of Veterinary Resources, Weizmann Institute of Science, Rehovot, Israel

**SUPPLEMENTARY INFORMATION**

**Day 6**

**T2-weighted**

**MT OFF**


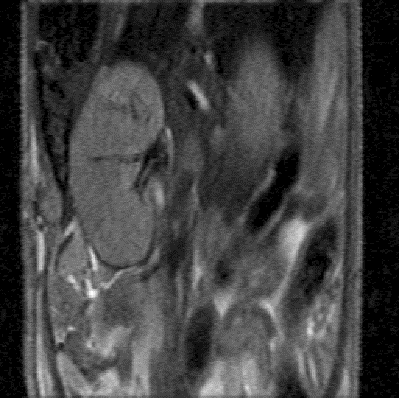

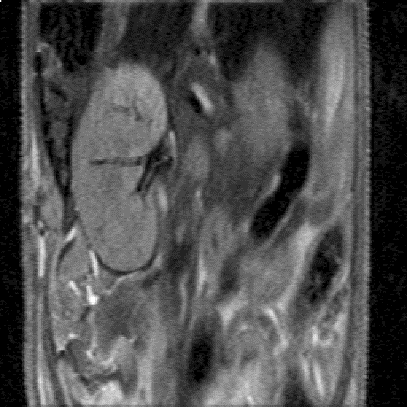

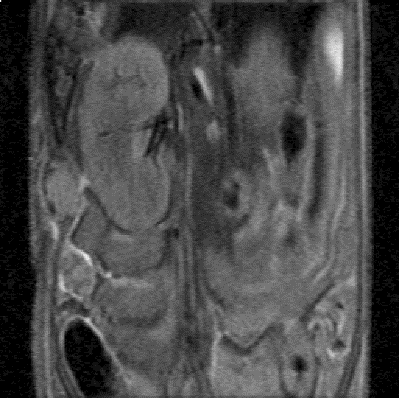

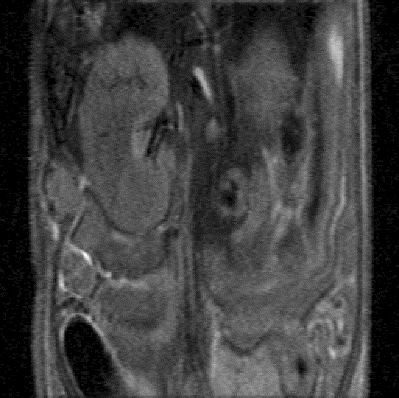

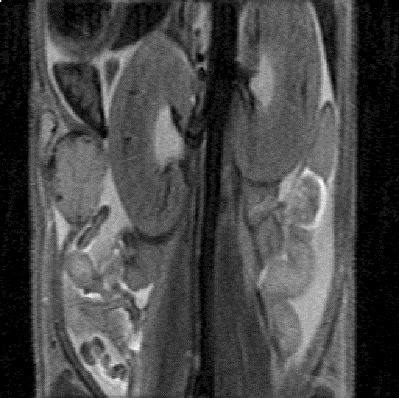

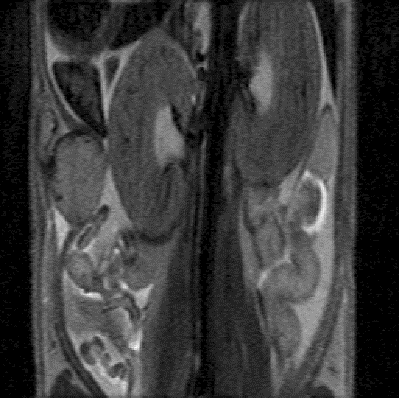

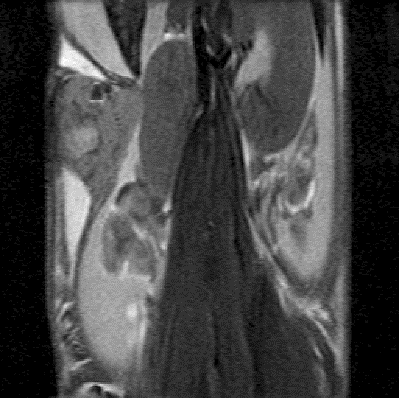

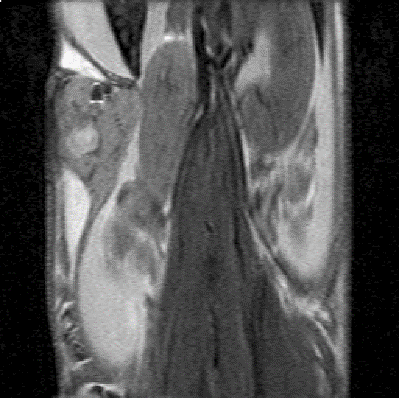


**Day 9**

**Day 12**

**Day 16**

**T2-weighted**

**MT ON**

**Supplementary Figure 1 | Abdomen of the CD1 nude mice at days 6, 9, 12 and 16 post-injection of ES2 cells into the ovarian bursa.** Orthotopic and skin lesion are marked with blue and red arrows respectively.


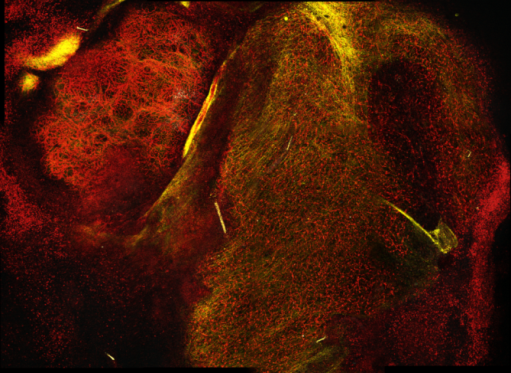


**OV**

**OV**

**FP**

**300 µm**


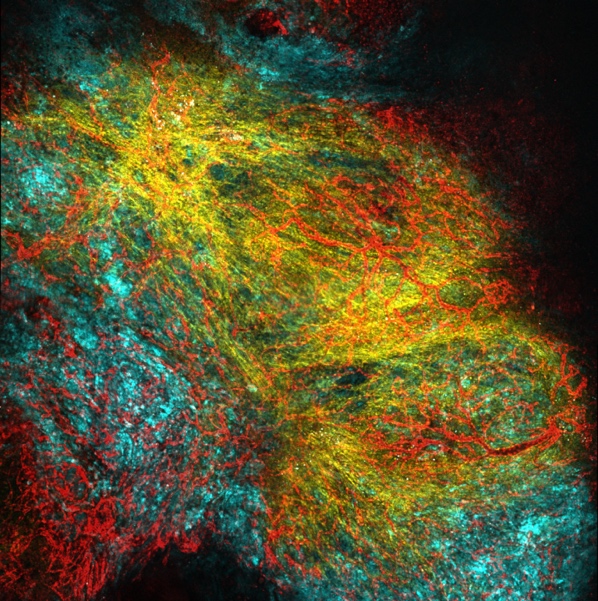


**200 µm**

**1**

**2**

**a**

**b**

**c**

**T**

**T**

**Supplementary Figure 2 |** **Ovary and fat pad of Vecad/tdTomato mouse affected with ID8 syngeneic tumor.** (a) Naïve ovary and fat pad unaffected by the tumor. (b) Fat pad 13 days post tumor grafting. (c) Fat pad 16 days post tumor grafting. Area 1 and 2 denote areas without and with abundant SHG signal, respectively. OV– ovary, FP – fat pad, T – tumor. Yellow – collagen (SHG), cyan – tumor cells (eGFP), red – endothelial cells (tdTomato).


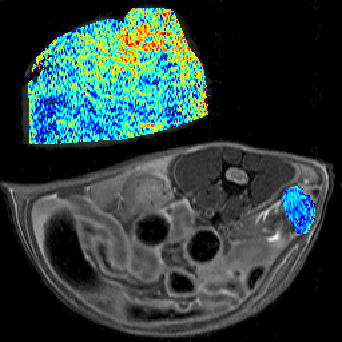

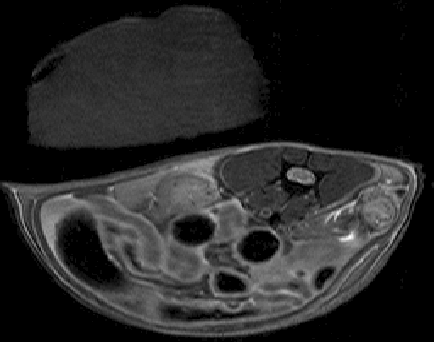

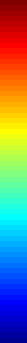


50%

40

30

20

10

0

**Day 64**

**a**


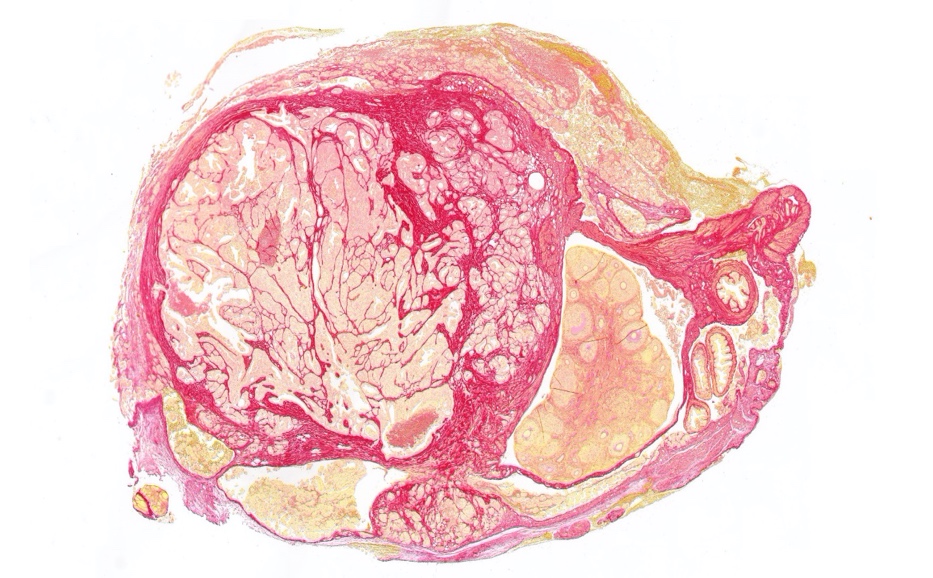


**1 mm**

**Day 79**

**b**

**MTR**

**Supplementary Figure 3 | Orthotopic PDX phenotype without the imaging window.** (A) T2 map and corresponding MTR maps of the PDX lesion at day 64 post tissue implantation. An agarose (2%) block was placed next to the animal to demonstrate that the protocol is sensitive for MT measurements. The tumor lesion is well separated from the surrounding tissue, similar to what was observed in the imaging window. (B) Sirius Red staining of the same lesion 79 days post tissue implantation.

|  | **M1** | **M2** | **M3** | **M4** | **M5** | **M6** | **M1** | **M2** | **M3** | **M4** | **M5** | **M6** |
| --- | --- | --- | --- | --- | --- | --- | --- | --- | --- | --- | --- | --- |
|  | **SL** | **SL** | **SL** | **SL** | **SL** | **SL** | **OL** | **OL** | **OL** | **OL** | **OL** | **OL** |
| **day 6** | **16.60** |  | **16.35** | **14.91** | **17.75** | **12.58** | **11.23** |  | **14.89** | **11.51** | **9.07** | **8.28** |
| **day 9** | **20.06** | **16.04** | **20.09** | **16.66** | **17.83** | **16.33** | **15.42** | **15.01** | **11.78** | **10.98** | **13.56** | **10.96** |
| **day 12** | **21.93** | **18.16** | **16.95** | **18.26** | **17.32** | **17.98** | **14.60** | **13.74** | **11.33** | **15.97** | **11.68** | **10.91** |
| **day 16** | **16.83** | **18.57** | **19.82** | **18.53** | **18.49** | **17.79** | **13.96** | **7.54** | **18.19** | **17.28** | **12.43** | **14.43** |

**Supplementary Table 1 | Mean MTR values [%] of skin lesions (SL) and orthotopic lesions (OL) throughout the longitudinal MRI experiment.**
